# Factors associated with burnout amongst healthcare workers providing HIV care in Malawi

**DOI:** 10.1371/journal.pone.0222638

**Published:** 2019-09-24

**Authors:** Maria H. Kim, Alick C. Mazenga, Xiaoying Yu, Katie Simon, Phoebe Nyasulu, Peter N. Kazembe, Thokozani Kalua, Elaine Abrams, Saeed Ahmed

**Affiliations:** 1 Baylor College of Medicine Children’s Foundation Malawi, Lilongwe, Malawi; 2 Baylor College of Medicine International Paediatric AIDS Initiative, Texas Children’s Hospital, Houston, Texas, United States of America; 3 University of Texas Medical Branch at Galveston, Galveston, Texas, United States of America; 4 Ministry of Health, Department of HIV and AIDS, Lilongwe, Malawi; 5 ICAP at Columbia University, Mailman School of Public Health, New York, New York, United States of America; 6 College of Physicians and Surgeons, Columbia University, New York, New York, United States of America; East Carolina University Brody School of Medicine, UNITED STATES

## Abstract

**Context:**

High rates of burnout have been reported in low and medium income countries and can detrimentally impact healthcare delivery. Understanding factors associated with burnout amongst health care workers providing HIV care may help develop interventions to prevent/treat burnout.

**Objectives:**

We sought to understand factors associated with burnout amongst health care workers providing HIV care in Malawi.

**Methods:**

This was a sub-study of a larger cross-sectional study measuring burnout prevalence amongst a convenience sample of healthcare workers providing HIV care in 89 health facilities in eight districts in Malawi. Burnout was measured using the Maslach Burnout Inventory. Anonymously administered surveys included questions about sociodemographics, work characteristics (work load, supervisor support, team interactions), depression, life stressors, assessment of type D personality, and career satisfaction. We performed univariable and multivariable regression analyses to explore associations between variables and burnout.

**Results:**

We received 535 responses (response rate 99%). Factors associated with higher rates of burnout on multivariable regression analyses included individual level factors: male gender (OR 1.75 [CI 1.17, 2.63]; p = 0.007), marital status (widowed or divorced) (OR 3.24 [CI 1.32, 7.98]; p = 0.011), depression (OR 3.32 [CI 1.21, 9.10]; p = 0.020), type D personality type (OR 2.77 [CI 1.50, 5.12]; p = 0.001) as well as work related factors: working at a health center vs. a rural hospital (OR 2.02 [CI 1.19, 3.40]; p = 0.009); lack of a very supportive supervisor (OR 2.38 [CI 1.32, 4.29]; p = 0.004), dissatisfaction with work/team interaction (OR 1.76 [CI 1.17, 2.66]; p = 0.007), and career dissatisfaction (OR 0.76 [CI 0.60, 0.96]; p = 0.020).

**Conclusion:**

This study identified several individual level vulnerabilities as well as work related modifiable factors. Improving the supervisory capacity of health facility managers and creating conditions for improved team dynamics may help reduce burnout amongst healthcare workers proving HIV care in Malawi.

## Introduction

Healthcare workers (HCWs) are the foundation of an optimally functioning health system, so when HCWs are not well, the performance of the health care system suffers [1, 2]. Burnout is a syndrome resulting from chronic job-related stress and has three characteristics: emotional exhaustion (physical or emotional depletion), depersonalization (negative or cynical feelings about patients) and a sense of low personal accomplishment (how one perceives one’s own competency) [3].

Burnout has negative impacts on patient care, HCW health, and the healthcare system. In terms of patient care, burnout is associated with medical errors, lower quality of care, and lower patient satisfaction [4–8]. In terms of HCW health, burnout can contribute to physical and mental illness, poor self-care and substance abuse [6–10]. Burnout may also lead to decreased HCW productivity and performance, higher HCW turnover, and absenteeism [7, 10–14]; all of which negatively impact and result in financial losses to the healthcare system.

HCWs in sub Saharan Africa (SSA) often work under burnout-inducing conditions: staffing shortages, rising numbers of patients, increasing burden of responsibility, low perceived control, unsupportive environments, staffing shortages, and the heavy weight of high rates of patient morbidity and mortality [15–17]. Research on burnout in SSA describe rates of 39–66% depending on country, HCW type, and survey instrument [18–22]. Despite these high rates, and the potential impact on patient care, there is a dearth of work examining factors associated with burnout in the SSA context; such research could inform the development of interventions to prevent and treat burnout and allay the negative downstream consequences of burnout.

In comparison, research describing HCW burnout in developed countries is firmly established, and the field has now largely moved towards examining potential interventions. In a recent comprehensive meta-analysis on burnout prevention and treatment interventions [21] only three of 52 studies included were conducted in low-income countries. That there were high rates of burnout amongst HCWs providing HIV care and that burnout was associated with a 3.2 times increased odds of reporting suboptimal patient care practices/attitudes [14].

There is a dearth of data on evidence-based culturally and contextually appropriate burnout reduction interventions. The present analysis seeks to address this gap by examining both individual and organizational/work-related factors that contribute to and protect against burnout. We hope that identification of these factors might inform the development of strategies to reduce burnout experienced by HCWs in Malawi who help care for an estimated number of 1,061,459 persons living with HIV every year [23].

## Methods

### Design and study setting

We analysed a sub-section of data that had been collected as part of a larger cross-sectional study conducted at 89 public health facilities within 8 districts in the south-eastern (districts: Balaka, Machinga, Mangochi, Mulanje, Phalombe, Zomba) and central regions (districts: Lilongwe, Salima) of Malawi from August 2015—January 2016 [14].

### Ethical approval

The National Health Sciences Research Committee in Malawi, as well as the Baylor College of Medicine (BCM) IRB in USA, granted ethical approval.

### Participants and data collection

The data collection procedures for this study have been previously described [14]. In brief, we approached a convenience sample of HCWs providing clinical care for people living with HIV (PLHIV) at 89 health facilities in central and southern Malawi to participate in the study. Of the 539 HCWs available and approached to participate, 535 (99%) consented and enrolled in the study. Participants completed self-administered written surveys. To reduce social desirability bias and encourage candid reporting the paper-based surveys were completed anonymously. As part of the consent procedure, participants were assured that their names would not be written anywhere on the surveys.

### Survey measures

Measures fully detailed previously [14] are described in brief below. Study measures not mentioned previously [14], are explained in detail below.

#### Burnout

We assessed burnout via the 22- question Maslach Burnout Inventory (MBI); a widely validated instrument for assessing HCW burnout [3, 18, 20, 24–29]. The MBI measures three constructs of burn out: Emotional Exhaustion (EE); Depersonalization (DP), to measure negative or cynical feelings about patients; and Reduced Personal Accomplishment (PA), to measure how one perceives one’s own competency. The larger study modified the MBI prior to data collection. The MBI was modified to improve clarity and relevance in the Malawi context. The standardized Cronbach alpha coefficients for the modified MBI were better [EE (0.75), DP (0.55), and PA (0.74), as compared to EE (0.67), DP (0.42), and PA (0.60)] than those found in a burnout study previously performed in Malawi [28]. We defined *burnout* as scores in the mid-high range on the EE (17–54) or DP (7–30) subscales based on cut-off scores used previously in Malawi [14, 28].

#### Depression, substance abuse, and personality type D

Depression was assessed using the World Health Organization’s (WHO) 20-item self-reporting questionnaire (SRQ) which had been translated and validated in Malawi, [30, 31]. A cut-off score of 8 was defined as a positive screen for depression. The standardized Cronbach’s alpha for the SRQ in this study was 0.79; comparable to prior research using the SRQ in Malawi (Cronbach’s alpha, 0.85) [31]. We also asked about prior history of depression.

The widely validated WHO Alcohol Use Disorders Identification Test (AUDIT) [32] was used to screen for at-risk use of alcohol. A total score of 8 was considered a positive screen for potentially hazardous or harmful use of alcohol [32–34]. To measure use of other recreational drugs, we asked, “How often do you use other drugs (marijuana, etc.),” and the score was adapted from the Drug Use Disorders Identification Test (DUDIT) [35].

Type D or “distressed” personality (TDP) is the tendency to experience a high amount of negative affectivity (NA) and social inhibition (SI) together [36]. TDP was assessed using the Type D Scale‐14 (DS14) which is the most accepted and widely used diagnostic instrument for the assessment of the TDP [37]. In prior studies, TDP has been found to be associated with burnout [36–39]. A cut off score of 10 (NA ≥10 and SI ≥10) was used to classify a participant as having TDP [37]

#### Socio-demographic and work characteristics

Participants’ self-reported demographics and work characteristics included: age, gender, marital status, number of children less than 5 years old, financial stress in the past 7 days. Work related demographic and characteristics included: HCW cadre, years worked as a HCW, type of health facility, number of hours worked in a typical week, time spent providing direct clinical care, number of patients seen in a typical week, adequacy of resources to provide quality care to patients, assessment of degree of support provided by the work environment and supervisor, need to perform work outside of regular duties to supplement one’s income, feeling that there are opportunities for growth, and satisfaction with work/team interaction (defined as ‘Yes’ if responding ‘Yes’ to the question ‘Do you enjoy your work-mates/colleagues?’ and ‘No’ to ‘Is there anyone at work who you have great difficulty working with?’. We assessed career satisfaction using three items modified from prior research) [40]: 1) Would you choose to become a health worker again? 2) Would you recommend to your children that they consider a career as a health worker? 3) If you could, would you switch to another career outside of health care? For the summary career satisfaction score we summed up the scores for all three questions. Each question had a binary score with 1 indicating ‘Yes’ and 0 indicating ‘No’. The score for question 3 was reversed before summing.

### Data analysis

Data were summarized by descriptive statistics (mean, SD, median, IQR, frequency). Chi-square test, two-sample t-test, and Fisher’s exact test were used to explore the associations between potential factors and burnout.

Logistic regression models were used to examine the association between burnout and individual level and structural/work related variables hypothesized to affect burnout based on prior work (socio-demographic and work-related demographics and characteristics, depression, alcohol/drug use and personality type D). We performed screening by univariable logistic regression. Variables were selected for inclusion in the model selection if their p-value was < 0.20 among the variables of interest. A backwards selection procedure was applied with a significance level of 0.05. Only variables with a p-value < 0.05 were retained in the final model. The scale for continuous variables was examined using quartiles to ensure a linear assumption was met prior to entry into the logistic model. The odds ratio estimates and their 95% confidence intervals were reported.

Missing items on the TDP and MBI were imputed using mean substitution for the same domain/subscale and the same participant [41–43]. Additional details can be found in the publication related to the larger study [14]. All analyses were performed using SAS software version 9.4 (SAS Institute, Inc., North Carolina, USA).

## Results

There were 535 HCWs enrolled in the main study. Of these, 15 completed surveys were excluded due to significant missing data. The mean age (SD) was 34 (10.2) years, 59% were female, 58% were married, 7% had a positive depression screen, 6% met criterion for at-risk use of alcohol and 62% met criteria for burnout. The majority (88%) provided clinical care more than 75% of the time, 71% felt they did not have adequate resources to provide quality care to patients, and 91% reported thinking additional HCWs were needed. A substantial proportion (36%) reported working more than 60 hours a week. We investigated financial stress and found that 87% reported feeling they needed to perform work outside of their regular duties to supplement their income and 79% reported stress due to their financial situation in the past week. In terms of career satisfaction, 88% reported that they would still choose to become a health worker again.

### Univariable analysis of associations between burnout and other variables

Variables associated with burnout on univariable analysis (Table 1) included the following socio-demographic or psycho-social variables: financial stress in the past 7 days, depression (as measured by the WHO SRQ, cut off ≥8), Type D personality. Age, gender, marital status, alcohol and drug use, were not found to be associated with burnout. The following work-related variables (Table 1) were found to be significantly associated with burnout: health facility type, number of patients seen in a typical day, reported need for additional staff, support from one’s supervisor, work relationships (enjoyment or difficulty with colleagues), and career satisfaction. The number of hours worked in a week, amount of time spent providing direct clinical care, feeling of control over work schedule/tasks, need to supplement one’s income and opportunities for growth were not found to be significantly associated with burnout on univariable analysis.

**Table 1:** Factors associated with burnout, univariable analysis.

Variable	Burnout No	Burnout Yes	p-value
**Participant sociodemographics, depression, and personality type D**			
**Age**, years, mean (SD)	33.7 (10.3)	33.8 (10.1)	0.948
**Gender,** n (%)			0.089
Male	73 (34)	142 (66)	
Female	126 (41.3)	179 (58.7)	
**Marital Status**, n (%)			0.075
Married	123 (40.9)	178 (59.1)	
Widowed/divorced	9 (22.5)	31 (77.5)	
Single	66 (37.1)	112 (62.9)	
**Children less than 5 years old**, n (%)			0.339
No	119 (36.4)	208 (63.6)	
Yes	76 (40.6)	111 (59.4)	
**Stress due to financial situation in the past 7 days**, n (%)			0.006
None	55 (49.5)	56 (50.5)	
Yes	144 (35.2)	265 (64.8)	
At-risk alcohol use (>/ = 8)* n (%)			0.331
No	189 (38.8)	298 (61.2)	
Yes	10 (30.3)	23 (69.7)	
**Other drug use,** n (%)			0.628
Never	197 (38.2)	319 (61.8)	
A few times a year	2 (50)	2 (50)	
**Depression- positive screen (>/ = 8)**, n (%)			0.002
No	194 (40.2)	289 (59.8)	
Yes	5 (13.9)	31 (81.6)	
**Suicidal Ideation**, n (%)			0.085
No	195 (39.1)	304 (60.9)	
Yes	4 (20)	16 (80)	
**History of Depression**, n (%)			0.072
No	194 (39.1)	302 (60.9)	
Yes	5 (20.8)	19 (79.2)	
**Personality Type D**, n (%)			<0.0001
No	181 (42.1)	249 (57.9)	
Yes	17 (19.1)	72 (80.9)	
**Work related demographics and environment**			
**Type of HCW**, n (%)			0.487
Medical Officer/Clinical officer/Medical assistant	69 (36.3)	121 (63.7)	
Nurse midwife technician/state registered nurse	130 (39.4)	200 (60.6)	
**Years worked as a health care worker**, median (IQR)	6 (3–10)	5 (3–11)	0.856
**Health facility type**, n (%)			0.035
District Hospital	54 (40.3)	80 (59.7)	
Rural Hospital	44 (48.9)	46 (51.1)	
Health center or other	101 (34.1)	195 (65.9)	
**Number of hours worked in a week**, n (%)			0.494
Less than 40 hours	12 (34.3)	23 (65.7)	
40–50 hours	94 (40.9)	136 (59.1)	
51–60 hours	28 (41.2)	40 (58.8)	
More than 60 hours	63 (34.2)	121 (65.8)	
**Time spent providing direct clinical care**, n (%)			0.820
All of my time	97 (39)	152 (61)	
>75%	77 (37.6)	128 (62.4)	
50%	16 (39)	25 (61)	
<50% or don’t provide clinical care	6 (28.6)	15 (71.4)	
**How many clients do you see in a typical day**, n (%)			0.024
< 25 hours	60 (47.2)	67 (52.8)	
25 to <50 hours	75 (40.5)	110 (59.5)	
50 to 100 hours	37 (34.6)	70 (65.4)	
>100 hours	22 (27.2)	59 (72.8)	
**Do you feel that you have adequate facility resources to provide quality care to patients**, n (%)			0.062
No	130 (35.7)	234 (64.3)	
Yes	69 (44.5)	86 (55.5)	
**Do you think your department needs additional members to accomplish your tasks,** n (%)			0.034
No	23 (53.5)	20 (46.5)	
Yes	175 (36.8)	300 (63.2)	
**How would you classify your work environment**, n (%)			0.077
Very supportive	26 (53.1)	23 (46.9)	
Supportive	141 (37	240 (63)	
Not supportive	31 (35.2)	57 (64.8)	
**Do you feel supported by your supervisor**, n (%)			0.016
Very supported	35 (53.8)	30 (46.2)	
Supported	139 (36.9)	238 (63.1)	
Not supported	24 (32)	51 (68)	
**How much control do you feel that you have over your work schedule and tasks**, n (%)			0.109
A lot	62 (34.4)	118 (65.6)	
Some	93 (37.2)	118 (65.6)	
Not much	34 (45.9)	40 (54.1)	
None	8 (61.5)	5 (38.5)	
**Do you need to perform work outside your regular duties to supplement your income,** n (%)			0.166
No	32 (45.7)	38 (54.3)	
Yes	166 (37.1)	282 (62.9)	
**Do you feel that you have opportunities for career growth,** n (%)			0.199
No	27 (31.8)	58 (68.2)	
Yes	168 (39.2)		
**Do you enjoy your workmates/colleagues,** n (%)			0.007
No	6 (16.7)	30 (83.3)	
Yes	190 (39.6)	290 (60.4)	
**Is there anyone at work who you have great difficulty working with,** n (%)			0.000
No	143 (44.4)	179 (55.6)	
Yes	55 (28.1)	141 (71.9)	
**Satisfied with work/team interaction,** n (%)			<0.0001
No	57 (27.5)	150 (72.5)	
Yes	139 (45)	170 (55)	
**Career Satisfaction**			
**Would you choose to become a health worker again,** n (%)			0.027
No	15 (25)	45 (75)	
Yes	181 (39.8)	274 (60.2)	
**Would you recommend that to your children that they consider a career as a health worker,** n (%)			0.066
No	172 (40)	258 (60)	
Yes	26 (29.5)	62 (70.5)	
**If you could, would you switch to another career outside of health care,** n (%)			0.002
No	164 (42.2)	225 (57.8)	
Yes	32 (26.2)	90 (73.8)	
**Sum of career items**			0.024
0	7 (18.4)	31 (81.6)	
1	11 (31.4)	24 (68.6)	
2	29 (34.5)	55 (65.5)	
3	147 (41.9)	204 (58.1)	
**Sum if career items,** mean (SD)	2.63 (0.75)	2.38 (0.99)	0.001

Analyses by chi-square test unless otherwise noted.

### Multivariable regression model: Factors associated with burnout

In multivariable analysis (Table 2), the following variables were found to be significantly associated with greater odds of burnout were: male gender, being widowed/divorced vs. being single or married (we did not find any statistically significant difference between being married vs. single), health facility type (working at a health center vs. rural hospital), depression, and personality type D. Those who reported having a very supportive supervisor and being satisfied with work/team interactions had lower odds of having burnout. With each unit increase in career satisfaction score, there was a 24% decrease in odds of having burn out.

**Table 2:** Final multivariable logistic regression model of factors associated with burnout.

Variable	OR [95% CI]	p-value	What does this mean?
Male vs Female	1.75 [1.17, 2.63]	0.007	Males were 1.75 times more likely to have burn out than females
Marital status			
Married vs. single	0.92 [0.60, 1.40]	0.694	No significant difference
Widowed/divorced vs. single	3.24 [1.32, 7.98]	0.011	Those who were widowed/divorced were 3.24 times more likely to have burn out than those who were single
Widowed/divorced vs. Married	3.52 [1.47, 8.47]	0.005	Those who were widowed/divorced were 3.52 times more likely to have burn out than those who were married
Health facility type			
District hospital vs. rural	1.47 [0.81, 2.67]	0.203	No significant difference
Health centre vs. rural	2.02 [1.19, 3.40]	0.009	Those working in health centre were 2.02 times more likely to have burn out than those working in rural hospital
Health centre vs. district hospital	1.37 [0.86, 2.18]	0.182	No significant difference
Support from supervisor			
Supportive vs. very supportive	2.38 [1.32, 4.29]	0.004	Those whose supervisor was supportive were 2.38 more likely to have burn out than those whose supervisor was very supportive
Not supportive vs. very supportive	2.34 [1.09, 4.99]	0.029	Those whose supervisor was not supportive were 2.34 more likely to have burn out than those whose supervisor was very supportive
Supportive vs. not supportive	1.02 [0.57, 1.81]	0.954	No significant difference
Satisfied with work/team interactions no vs yes	1.76 [1.17, 2.66]	0.007	Those not satisfied with their work/team interaction were 1.76 times more likely to have burn out than those who were satisfied
Depression score ≥8	3.32 [1.21, 9.10]	0.020	Those with depression score ≥8 were 3.32 times more likely to have burn out than those with depression score <8
Type D personality, yes vs no	2.77 [1.50, 5.12]	0.001	Those with type D personality were 2.77 times more likely to have burn out than those without type D personality
Career satisfaction	0.76 [0.60, 0.96]	0.020	With each unit increase in career satisfaction score, there was a 24% decrease in odds of having burn out.

The c-statistic for this model is 0.71 indicating good discriminatory ability of the model; the value can range from 0 to 1 with a higher value indicating better discriminatory ability. The p-value of Hosmer-Lemeshow Goodness of fit test was 0.22 which is not significant (p>0.05) demonstrating no lack of fit; in other words, the overall fit of the model was good.

## Discussion

The primary goal of this study was to facilitate the development of burnout reduction interventions for healthcare workers in low resource settings like Malawi by identifying factors that might be amenable to interventions and were associated with burnout. The present study identified several characteristics that were significantly associated with burnout amongst health care workers providing HIV care. At the individual level factors included male gender, marital status (widowed/divorced), and depression. Work related factors included working at a health center vs. a rural hospital, lack of a very supportive supervisor, dissatisfaction with work/team relationships, and career dissatisfaction.

The HIV treatment response in Malawi has resulted in an enormous influx of additional patients into the healthcare system creating mounting demands on an already under-resourced system and its HCWs. Malawi’s total expenditure on health as a percentage of GDP is the highest in the Southern African Development Community (SADC) [44], perhaps suggesting the government’s commitment to healthcare. Unfortunately, despite this, Malawi’s healthcare system is highly underfunded, with the lowest health spending per capita in the SADC due to the very low GDP per capita of only $339 USD. Donor support provides close to 80% of the healthcare expenditure, and has become more and more fragmented and increasingly focused on treating specific diseases [45]. The disease specific donor funding, often neglects the basic primary health care systems and human resources that are the essential foundation required to achieve optimal health outcomes.

In the context of these considerable structural limitations, there is an urgent need for strategies focused on strengthening the healthcare workforce by preventing/managing HCW burnout. The situation is grave. Studies on HCW burnout from sub-Saharan African countries including Malawi, report burnout rates ranging from 33–66% [9, 14, 18, 19, 22, 25, 28], and burnout can have destructive implications for patient outcomes. For example, in Malawi, there has been an increase in the number of individuals living with HIV who are not retained in ART care. Currently, in Malawi only 72% of adults and 77% of children were retained alive on ART 12 months after ART initiation [23]. The poor retention rate could be a downstream effect of suboptimal patient care practices resulting from HCW burnout [14]. Burnout can result in acrimonious relationships with patients. Malawian and South African patients have reported that conflicts with providers led some to stop coming to clinic visits, eventually abandoning clinic care [46, 47].

Our present study identified several areas at both the individual and organizational/work related level, that are amenable to interventions. Finding the resources to develop and deliver these interventions will be challenging but our study suggests that the time is ripe. Although burnout was associated with decreased career satisfaction, the majority of HCWs reported that they would still choose to become a HCW again. This suggests dedication and interest in this career path; and therefore, HCWs that would be eager to receive burnout reduction support.

Addressing HCW burnout is a shared responsibility between individual HCWs and work organizations, and therefore requires both individual (HCW) level and organizational/work-related interventions [8]. We identified specific individual level factors associated with high rates of burnout including gender, marital status, depression, and personality type D; these findings are consistent with studies from other countries [36, 39, 48–50]. This was a cross-sectional study, so we are unable to determine causality. For example, did depression contribute to vulnerability to burnout? Or did burnout lead to depression? However, our data suggest that men, those who are widowed/divorced, are depressed, or have personality type D may be more vulnerable to burnout. Individual level interventions that have been shown to be effective in reducing burnout in other settings include mindfulness based stress reduction, stress management training, therapy, as well as small-group activities with other HCWs to share work experiences [8]. Many of these interventions are feasible even within a resource-limited setting and should be explored.

Organizational interventions have high potential to ease burnout [8]. We identified modifiable work related characteristics associated with burnout that could be improved by health facility leadership and Ministry of Health. HCWs working at district hospitals and non-rural health centers had higher odds of burnout. This may be due to stresses of working at these typically higher volume, sub-optimally staffed facilities. Consistent with HCW burnout studies from other countries, having very supportive supervisors and positive work/team engagement was protective [9, 51]. Ill prepared clinicians without management experience or training are often hoisted into supervisory roles [52]. Leadership/management training could equip clinician supervisors to provide more supportive supervision, tackle challenging employees who may be negatively affecting team morale, and craft healthier work environments.

Although financial stress was not associated with burnout in the final MVLR model, we did find a significant bivariate association. In addition, the majority (86%) reported financial stress in the past week as well as the need to work outside their clinical duties to supplement their incomes (86%). Inadequate HCW compensation has been a chronic challenge in resource limited settings [53–57]. Although in the short run such costs may seem prohibitive, adequate salaries may reduce absenteeism (because HCW will be less inclined to seek additional paying work outside the clinic), as well as burnout, resulting in improved patient outcomes and cost savings.

Despite an urgent need to address burnout amongst HCWs in Malawi, unfortunately, we are not aware of any burnout reduction or prevention interventions that have been developed, piloted, tested, or implemented in Malawi. In contrast, there is an abundance of research on HCW burnout interventions in the northern hemisphere demonstrating that both individual-focused and structural or organizational strategies can result in clinically meaningful reductions in burnout [17, 58]. However, it is unclear whether or not these same types of interventions will work in the Malawian context. There is a paucity of evidence on potential burnout reduction interventions from resource limited settings. By identifying both individual and structural level factors that could inform intervention development, our study represents an initial step towards addressing this gap.

Our study had a high response rate and although self-reported surveys may be inclined to social desirability bias, the surveys were conducted anonymously to try and reduce this bias. In addition, surveys were conducted in both rural and urban settings and throughout central and southern Malawi and therefore, the results are likely representative of HCWs providing HIV care in most of Malawi. We explored various factors that have been linked to burnout in other countries, and although we did not utilize more comprehensive scales due to length [59], we explored work-related and organizational characteristics thought to contribute to burnout such as work team dynamics, organizational constraints, workload, lack of control in the work environment, and opportunities for career growth. Surprisingly, hypothesized contributors to burnout such as workload, lack of opportunities for career growth, and lack of control in the work environment [60–62], were not found to be linked to burnout in our study. This might be because we assessed these characteristics using single questions, which perhaps did not sufficiently assess these characteristics. In future studies, it would be helpful to use more comprehensive scales such as the five-item Quantitative Workload Inventory [59] to further explore these hypothesized burnout contributors.

## Conclusion

In summary, we identified several factors associated with burnout amongst health care workers providing HIV care. Individual level factors included male gender, marital status (widowed/divorced), and depression. Work related factors were working at a health center vs. a rural hospital, lack of a very supportive supervisor, dissatisfaction with work/team interaction, and career dissatisfaction. Our results underscore the critical need for strategies to prevent/manage HCW burnout in Malawi, and identify both individual and organizational level characteristics that future interventions could address.
